# Antimicrobial Peptides as Therapeutic Agents

**DOI:** 10.1155/2012/326503

**Published:** 2012-06-19

**Authors:** Mathew Upton, Paul Cotter, John Tagg

**Affiliations:** ^1^Microbiology and Virology Unit, School of Medicine, University of Manchester, Oxford Road, Manchester M13 9PL, UK; ^2^Teagasc Food Research Centre, Alimentary Pharmabiotic Centre, Moorepark, Fermoy, Cork, Ireland; ^3^Department of Microbiology and Immunology, University of Otago, P.O. Box 56, Dunedin, Otago, New Zealand

In developing this special issue, we sought to raise awareness of the potential of antimicrobial peptides (AMPs) for clinical use and to highlight recent developments in the field. The issue contains research articles covering the full spectrum of contemporary work, ranging from the rational design of synthetic peptides and discovery of bacteriocins, to testing of the efficacy of AMPs in animal models. We have included two expert reviews reflecting on the potential of both eukaryotic and prokaryotic peptide antibiotics for clinical use.

The antibiotic development pipeline is devoid of imminent solutions to the problems caused by resistant gram-negative bacteria and only a small number of new agents exist for therapy of infections caused by gram-positives. The pathway for development of new antibiotics is both long and extremely expensive and, therefore, “big-pharma” has not invested significantly in this area for decades.

The scarcity of antibiotics to combat infectious disease is commonly acknowledged and the specter of untreatable infections has been widely publicized. However, some additional potential impacts of the absence of efficacious antibiotics are not as widely discussed. The inability to reliably treat infections could potentially mean that all elective surgery ceases, and, moreover, there could be significant restrictions placed on the use of cancer chemotherapy. Thus, in order to maintain anything like the level of medical care that many of us now enjoy, we must devote considerable effort to find solutions to this steadily-evolving clinical dilemma. AMPs appear to merit a prominent position in the catalogue of pharmaceuticals that should be more exhaustively investigated in our search for new antimicrobial agents.

Many AMPs have potent activity against bacteria, including those that are resistant to conventional antibiotics. Their activity is often relatively-specifically directed against certain genera or groups of bacteria, which could limit damaging effects on a patient's commensal flora. AMPs generally exhibit high stability to wide ranges in pH and temperature, characteristics that may be beneficial for their scaled-up production and formulation into deliverable products. Also, due to their rather specific modes of action, many AMPs exhibit low toxicity for eukaryotic cells, providing an opportunity for a wide therapeutic window. Their typical mode of action is also linked to a low propensity for resistance development in target bacteria. These unique features differentiate AMPs from many conventional antibiotics. 

Outside of the potential use of AMPs as alternatives to antibiotics with respect to the treatment/prevention of disease, numerous recent reports have also described the activity of AMPs against bacteria growing in biofilms, that is, communities encased in structured matrices of extracellular macromolecules, on medical devices. Medical devices can become colonized with bacteria growing in biofilms, which are resistant to elements of the host immune response and to extremely high concentrations of conventional antibiotics. Consequently, resolution of medical device-related infections usually requires the removal of the device. Notably, low concentrations of some AMPs have been shown to have inhibitory and disruptive properties that can eliminate even well-established biofilms.

It is also important to consider the fact that some AMPs have been shown to exhibit immunomodulatory properties; that is, they can reduce the tissue-damaging inflammatory response to infection, whilst stimulating antimicrobial immune activities. Peptides exhibiting modulatory features, as well as overt antimicrobial activity, are now being selected as favoured candidates for further evaluation in clinical trials.

Recently, the search for AMPs has benefited from a number of advances in informatics and peptide engineering strategies. This, coupled with renewed interest from big-pharma, manifested by some recent acquisitions of companies using natural product screening approaches, is increasing the prominence of AMPs. This current special issue includes research articles and reviews that together describe the process of discovery of AMPs, their testing in *in vivo* models and outlines steps taken in the development of clinically-relevant agents.

The paper by H. Jin-Jiang and colleagues describes the design and synthesis of a range of AMPs and reports on the activity of K11, a 20 mer highly cationic helical peptide with activity against gram-positive and gram-negative bacteria, including drug resistant clinical isolates. K11 has a high therapeutic index/window and clearly warrants further investigation. Thus, the paper demonstrates the utility of synthetic approaches to the development of novel chimeric AMPs.

Also included in the special issue is a report by P. A. Wescombe and colleagues, which focuses on the characterization of a bacteriocin from the SA-FF22 family produced by members of the *Streptococcaceae*. The plasmid-encoded peptide G32 was purified from a culture of *Streptococcus salivarius*, a species known to include numerous bacteriocin producers, and is active against the pathogen *Streptococcus pyogenes*. An interesting feature of this lantibiotic peptide is the apparent absence of an auto-induction capability. The work supports previous suggestions that *Streptococcus salivarius* mega-plasmids are repositories for lantibiotic loci and that bacteria producing these lantibiotics can be effective probiotics.

C. Piper and colleagues demonstrate the utility of whole animal bioimaging approaches to demonstrate that the lactococcal lantibiotic lacticin 3147 could prevent systemic spread of *Staphylococcus aureus* following subcutaneous delivery of the peptide. This is important work as it adds to the growing evidence base that suggests that lantibiotics and other AMPs could potentially be used to control systemic infections, most previous approaches having been restricted to topical applications.

The two reviews included in this issue elegantly précis recent advances that have been made with AMPs derived from both bacteria and insects. M. Ntwasa and colleagues describe the medical potential of immune system peptides of the coleopteran insects. The authors report that these ancient eukaryotes have a highly developed and robust immune system that has contributed to their survival. Exploitation of the immune effectors from these organisms holds great potential for use in clinical medicine. The review also describes the utility of databases and full genome sequence determination in AMP discovery.

The paper by C. T. Lohans and J. C. Vederas is an extensive review of the potential utility of the unmodified, or class IIa, bacteriocins. The paper describes peptide engineering approaches for increasing the value of bacteriocins, methods for improved *in vitro* production and efficient purification, various *in vivo *delivery options and, finally, gives some attention to the important issue of the development of resistance to bacteriocins. The paper concludes with a positive outlook for the use of bacteriocins in medical therapy.

We agree that there is great potential for the use of AMPs in clinical therapy for infectious disease, either through direct antimicrobial activity or modulation of the immune response to infection. Contributors to this special issue also discuss the possibility for use of some AMPs in antitumor therapy. AMPs offer some significant advantages over conventional antibiotics and we are now making important advances in the discovery, optimization, and activity testing of these agents. In parallel, publications in this field are attracting the attention of increasing numbers of academic and industrial researchers. It is genuinely an exciting time to be involved in the development of AMPs and we trust that this special issue leaves readers similarly enthused about the subject.

*Mathew Upton* *Mathew Upton* *Paul Cotter* *Paul Cotter* *John Tagg* *John Tagg*

